# Recurrent Hemoptysis in an Adult Patient With an Unfamiliar Cause

**DOI:** 10.7759/cureus.36062

**Published:** 2023-03-13

**Authors:** Anshuman Darbari, Mayank Mishra, Russel Abisho, Udit Chauhan, Deepansh Gupta

**Affiliations:** 1 Cardiothoracic and Vascular Surgery, All India Institute of Medical Sciences, Rishikesh, Rishikesh, IND; 2 Pulmonary Medicine, All India Institute of Medical Sciences, Rishikesh, Rishikesh, IND; 3 Radiology/Interventional Radiology, All India Institute of Medical Sciences, Rishikesh, Rishikesh, IND

**Keywords:** bae, cyanotic heart disease, adult cardiac disease, tetralogy of fallot, hemoptysis

## Abstract

Most adult patients who experience recurrent hemoptysis have respiratory or coagulopathy-related causes and cardiac aetiology in very few cases. In this rare case of 56 years aged male patient who presented to us with chronic recurrent hemoptysis, Tetralogy of Fallot (TOF) was the culprit aetiology, and he was successfully managed by minimal intervention.

## Introduction

Tetralogy of Fallot (TOF) is a cyanotic congenital heart disease with an incidence of 0.34 per 1000 live births and is primarily diagnosed in childhood [1]. Rarely does it become unnoticed, and the patient may remain asymptomatic and diagnosed later [2]. Some cases are also incidentally diagnosed later in the routine investigation or may be diagnosed with atypical presentation. Here, we present a case of an adult male patient in his late 50s who presented with recurrent hemoptysis. On evaluation, he was found to have TOF with major aortopulmonary collateral arteries (MAPCAs) and later managed successfully.

## Case presentation

**Figure 1:**
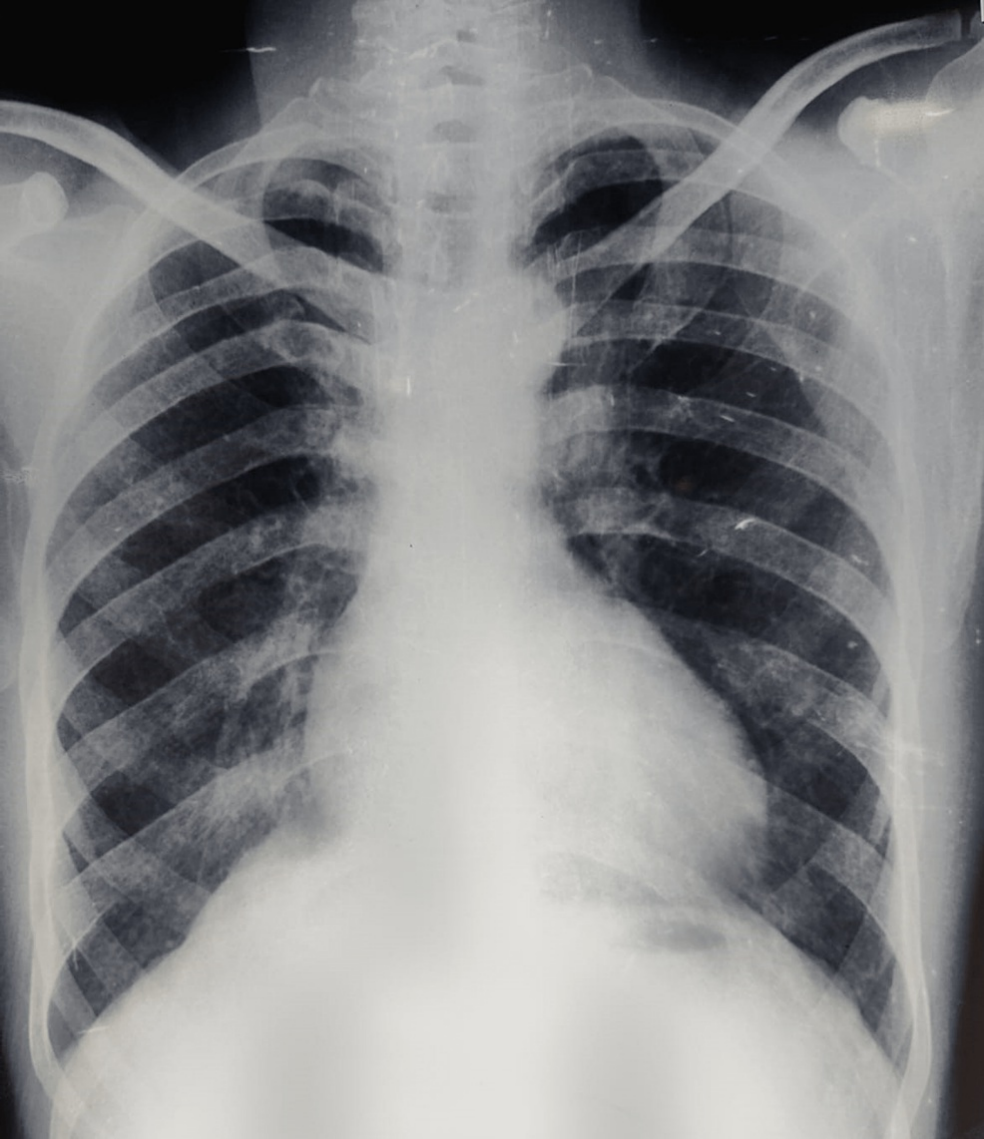
Chest X-ray PA view showing mild cardiomegaly with right ventricular type apex. PA = posteroanterior

A medium-built male of 56 years of age from a remote, rural area presented to us with complaints of recurrent episodes of mild hemoptysis for 20 years, which he regarded as minor. The bleeding has suddenly increased in the past two weeks and is now up to approximately 100 ml each day. He was also having insidious onset history of shortness of breath and mild chest pain for the last three weeks. He was a non-smoker, non-diabetic and without any other significant medical history. According to the patient and the attendant, there was no history of cyanosis or cyanotic spells. During clinical examination, there was slight pallor, significant cyanosis in the nail bed and lips, and grade-4 clubbing of all the digits. In-room air, peripheral saturation was 85%. Cardiovascular examination showed palpable thrill along the left sternal border, the first heart sound was normal, and the second heart sound was single (P2 component was absent) with grade-3 ejection systolic murmur at the left sternal border and second intercostal space. Mild splenomegaly was revealed on abdominal examination. Baseline blood investigations showed low haemoglobin (Hb) value of 7.0, but other parameters and coagulation profiles were in the normal range. Chest radiography showed a boot-shaped heart and mild cardiomegaly with a right ventricular-type apex (Figure 1).

**Figure 2:**
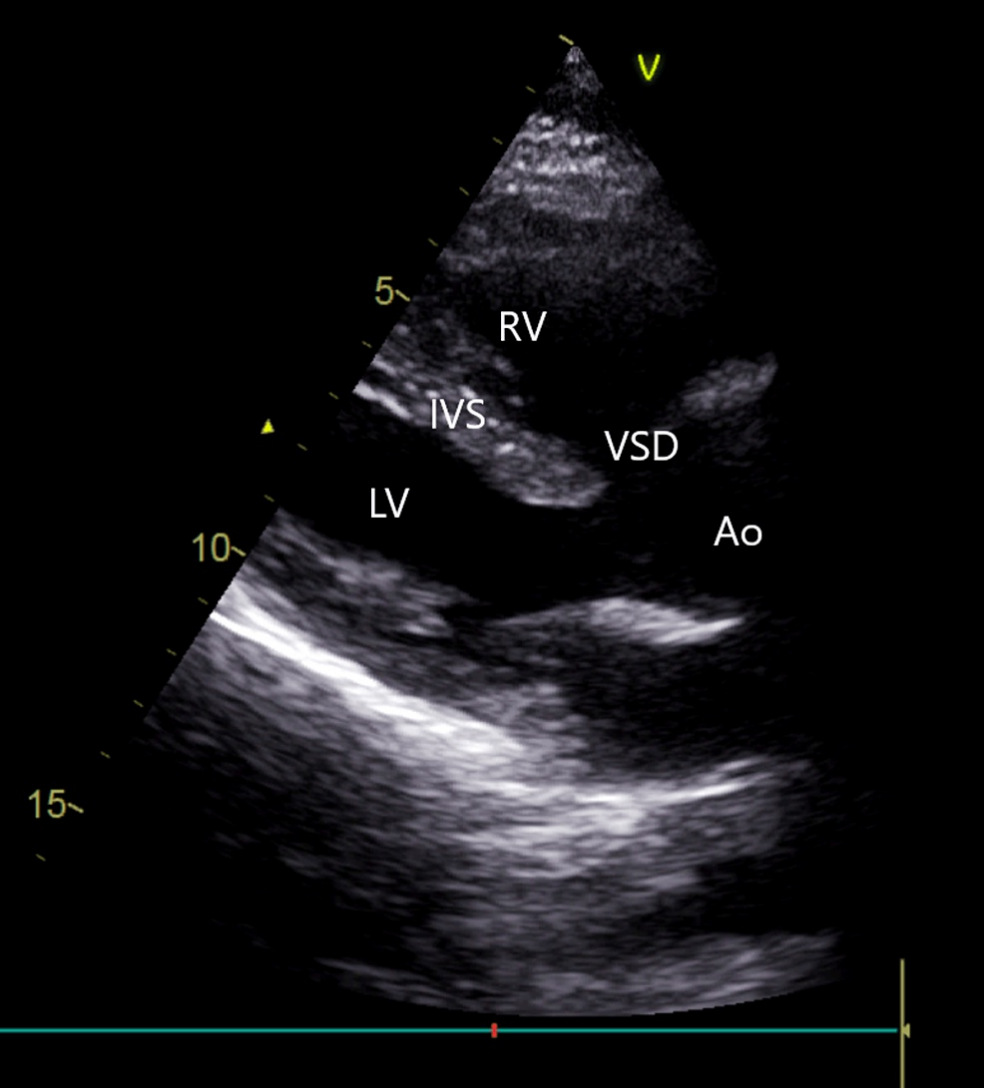
Transthoracic echocardiography showing large sub-aortic ventricular septal defect with overriding of the aorta with severe valvular pulmonary stenosis and right ventricular hypertrophy. RV = right ventricle, LV = left ventricle, IVS = interventricular septum, VSD = ventricular septal defect, Ao = aorta

2-D transthoracic echocardiography showed a large subaortic ventricular septal defect with overriding of the aorta and near equalization of shunt flow with severe valvular pulmonary stenosis, moderate tricuspid regurgitation, with normal biventricular function (Figure 2).

**Figure 3:**
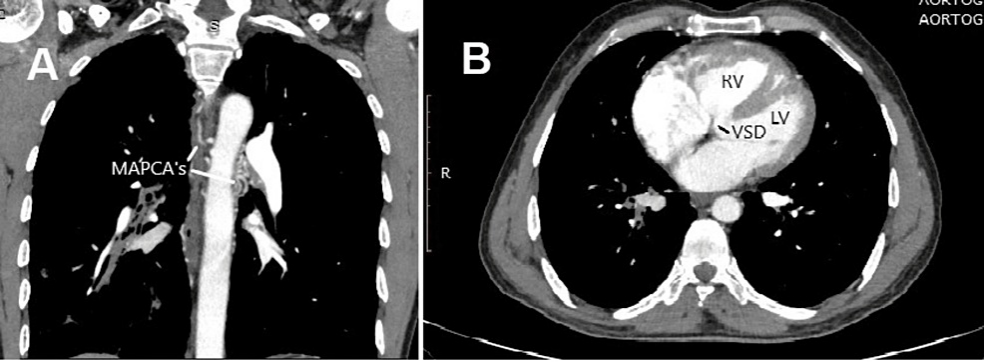
Cardiac computed tomography showing (A) multiple aortopulmonary collateral arteries (MAPCAs), (B) ventricular septal defect with overriding of the aorta, right ventricular hypertrophy. RV = right ventricle, LV = left ventricle, VSD = ventricular septal defect

Cardiac computed tomography (CT) scan showed situs solitus, significant right ventricular outflow tract obstruction, thickened right ventricle, large membranous ventricular septal defect of 14.4 mm with overriding of the aorta, dilated right and left bronchial arteries with MAPCAs at 2’0 clock and 11’0 clock. These investigations confirmed the pathoanatomical diagnosis of TOF physiology with MAPCAs (Figures 3A-3B).

After all the necessary relevant cardiac investigations as described above, flexible bronchoscopy was done, which showed old clots in the right lower lobe bronchus. Bronchial alveolar lavage was also done, and conservative measures were started. The patient was advised for coronary angiography and cardiac catheterization study to further plan of corrective surgery. But he and his attendants refused surgery, and any further intervention considering the risk and his age. So bronchial artery embolization (BAE) was offered as a less invasive choice. They accepted this intervention after perusal and explanation. In the BAE procedure, after passing the standard guide wire through the femoral route super selectively embolization of all anomalous vessels, mainly the right-sided bronchial artery, and both MAPCAs were done. The procedure was done by a 2.7 Fr microcatheter using polyvinyl alcohol (PVA) material in the culprit's vessels to achieve hemostasis. The patient was relieved of hemoptysis and was symptom-free at the time of discharge after three days of BAE. At the time of discharge, he was prescribed lanoxin, loop diuretics, with propranolol (non-selective beta-blocker) in standard doses to prevent right ventricular failure and to reduce right ventricular infundibular spasm. In follow-up, the patient is free of hemoptysis, and anaemia is corrected without other blood parameter disturbances. He is able to do his normal day-to-day activities with minimal exercise intolerance on prescribed drug therapy.

## Discussion

TOF is the most common cyanotic heart disease with an abysmal prognosis if left uncorrected. Children with TOF are mainly present in the first year of life with cyanotic spells or failure to thrive. Once diagnosed, early surgery is recommended for a better outcome. Lillihei et al. did the first successful corrective surgery in 1955 [3]. In some cases, TOF patients remain asymptomatic till later age. Survival of uncorrected TOF is rare; only 10% reach age 25, and only 2% reach over 40 [4,5]. Survival depends on the severity of pulmonary stenosis and the development of systemic-pulmonary collateral circulation [6].

Adult TOF rarely remains without symptoms, or symptoms may be neglected, as in our case. In our case, the patient had hemoptysis for a long time. Still, it was ignored, and the patient survived till now due to multiple MAPCAs and the gradual development of pulmonary stenosis. Possible reasons for long-time survival include gradual development of pulmonary stenosis giving time for systemic-pulmonary collateral formation, degree of stenosis and collateral formation and reduced left ventricular compliance. Pulmonary atresia patients have less chance of survival than pulmonary stenosis cases [6,7].

Adult TOF may present with hemoptysis, epistaxis and rarely with cerebrovascular accidents or infective endocarditis. Adult TOF repair has an increased risk of postoperative complications compared to early repair. Early postoperative complications include excessive bleeding due to well-formed collaterals, coagulopathy, arrhythmias and heart block [8,9]. Later complications include right ventricular dysfunction, congestive heart failure, tricuspid regurgitation and pericardial effusion. Even with better surgical techniques and postoperative care, mortality and morbidity in adult TOF repair are relatively high [10].

Very few case reports of these uncorrected TOF in an adult patient are present in the medical literature [11,12]. As our patient denied surgery and he was hemodynamically stable, hemoptysis was treated by BAE and continued on conservative medical management. In this case, even corrective surgery for TOF may not have relieved this unusual symptom.

## Conclusions

This case is a rare presentation of uncorrected TOF in an adult patient with an unusual presentation of hemoptysis. Thorough cardiac evaluation by 2-D echocardiography followed by a cardiac CT scan may be fruitful for this kind of rare presentation of TOF. The elderly with asymptomatic TOF with anomalous collaterals or MAPCAs can be managed medically or by selective embolization. Corrective surgery for TOF in the elderly is still debatable. Henceforth, if the patient is asymptomatic in a case of elderly TOF, corrective surgery may be avoided and it can be treated symptomatically.
